# Phytochemicals and antioxidant activity of leaf extract and callus cultures of *Cinnamomum camphora* L

**DOI:** 10.1371/journal.pone.0321155

**Published:** 2025-11-14

**Authors:** Sajal Rasool, Kainat Rasool, Sheza Ayaz Khilji, Zahoor Ahmad Sajid

**Affiliations:** 1 Institute of Botany, University of the Punjab, Quaid-e- Azam Campus, Lahore,; 2 Department of Botany, Ghazi University, Dera Ghazi Khan, Pakistan; 3 Department of Botany, Division of Science and Technology, University of Education, Lahore; Srimad Andavan Arts and Science College, INDIA

## Abstract

*Cinnamomum camphora* L. is highly significant landscape tree known for its medicinal values and presence of secondary metabolites that have antioxidant, antimicrobial, anticancer, anti-inflammatory effects and widely utilized in pharmaceutical and cosmetic industry. Callus cultures of *C. camphora* have better antioxidant activity than extracts of naturally grown tree leaves. While there is significant lack of research about the potential of its *in vitro* callus cultures as a controlled and sustainable biotechnological alternative for mass production of bioactive compounds. The present work was aimed at comparative analysis of phytochemicals (phenolic and flavonoid) and antioxidant activities of calli and leaf extract of field grown camphor plant. To get *in vitro* germplasm, callus formation and direct shoot initiation was carried out and it was observed that MS medium supplemented with 0.1 mg L^-1^ thidiazuron (TDZ) + 0.5 mg L^-1^ 2, 4-dichlorophenoxyacetic acid (2, 4-D) proved best for shoot initiation from nodal explant. MS medium fortified with various plant growth regulators was used for callus formation and best callus induction response (100%) from nodal and leaf explants was observed on 0.5 mg L^-1^ 2, 4-D) + 2.0 mg L^-1^ 6-Benzylaminopurine (BAP). Callus was successfully sub-cultured and this *in vitro* proliferated calli and fresh leaf extract of field grown plant were used for comparative study of phytochemicals. Results revealed that callus culture exhibited highest antioxidant enzyme activities compared to leaf extract and hence there was statistically significant (*P ≤ 0.05*) difference. Similarly, peroxidase (31.12 UmL^-1^ of enzyme), superoxide dismutase (35.24 Umg^-1^ of protein), and catalase (58.6 UmL^-1^ of enzyme) also showing highest vales. In contrast, glutathione peroxidase activity (0.552 Umg^-1^) was comparatively higher in leaf extract. Additionally, callus cultures accumulated higher phenolic contents (1.106 mg GAE g^-1^ of FW) while flavonoid contents (7.87 mg CatE g^-1^ of FW) were higher in leaf extract. This investigation showed that *in vitro* conditions and the use of plant growth regulators in various combinations might be work as elicitors to enhance the phytochemicals and antioxidant enzymes in callus culture as compared to the leaf extract.

## Introduction

Phytochemicals are the most significant group of secondary metabolites that are produced by plants for their reproduction, symbiotic relationships, growth and development [1]. Although they are already present but stressful growth environments or modifications to the growth medium may further trigger their synthesis like plant grown under in *vitro* conditions [2,3]. The majority of these chemicals are produced constitutively but their synthesis can be increased under stress in a way that it depends on the growth circumstances and the stressor [4,5]. Phenolic content and antioxidant activity in plant products have been shown to be positively correlated with each other [6]. One of the major subgroups of phytochemicals with potential antioxidant benefits and positive impacts on human health is the polyphenol [7] that have significant role in determining the flavour, texture, color, and sensory perception of food [8,9]. Phenolics are the most promising phytochemical for further studies that have vital role in the detoxification of H_2_O_2_ in plants, as well as in UV protection and enzyme modulation [10,11]. Some researches claim that the anticancer properties of phenolic compounds are associated to apoptosis [12]) and the identification with standardization of phytochemicals play crucial role for its effective treatment [13].

Similarly, flavonoids are actively present polyphenolic phytochemicals secreted by plants [14] that have been utilized in a variety of herbal remedies since ancient times as they have antiviral, anti-bacterial, anti-inflammatory, anticarcinogenic and antimutagenic properties [15]. Phenylpropanoid pathway synthesizes flavonoid [16], while they are synthesized in response of any microbial infection or disease and mainly accumulated in the edible sections of the plants [17]. There are many studies in literature, conducted on various medicinal plants to explore the phytochemicals and their pharmaceutical effects on diseases. Traditional medicines made from a variety of medicinal plants are widely used in countries; Pakistan, China, India, Bangladesh, Korea, Taiwan, Japan, and Sri Lanka. Among many of medicinal plants *C. camphora* bearing with high significance because of its herbal and ritual use especially in China and Sub-continent.

*Cinnamomum camphora* L. Presl is a perennial tree that belongs to the family *Lauraceae*. It is naturally cultivated as a landscaping tree, and is used as an important herbal medicine in southern China [18,19]. It is also known as Camphor tree, Camphor, Camphorwood or Camphor laurel, and found in temperate to subtropical regions of East Asian countries, i.e.*,* China, Japan, Korea, Vietnam, especially along the coast from Cochin China (Vietnam) and also belongs to the estuary of the Yangtzekiang (river), adjoining islands; Hainan and Taiwan and the naturally grown range bounds to just about 10–36°N and 105–130°E [20]. Leaf essential oil (CEO) has potent antioxidant, antimicrobial, anticancer, strong insecticidal [21] and anti-inflammatory effects [22]. The CEOs are being widely utilized in pharmaceutical, food and cosmetics industries as significant raw resources [23]. Camphor is helpful in evaluating more possible pharmaceuticals and creating new anti-inflammatory medications [24]. In Chinese tradition, camphor leaves may treat gastrointestinal problems like diarrhea as well as mental problems like hysteria, anxiety, and neuralgia [25]. Further, various species of the genus *Cinnamomum* are also utilized as significant condiments [26]. According to some studies, ethanolic extract from leaves of camphor plant is significantly effective in treating atopic dermatitis [27]. It has been showed that leaves of *C. camphora* have remarkable anti-inflammatory capability in adult human [28]. Extracted essential oils from various parts of *C. camphora* such as twigs, leaves, and seeds, has notable insecticidal potential, leaves essential oil is additionally used as fruits and vegetables preservative and is predicted to have a lot of other applications. In future, the researcher hopes to develop bio-based products by using *C. camphora* leaves in medicine industry [29].

Besides this huge pharmacological, industrial and environmental significance, *C. camphora* is not propagated at large scale in Pakistan due to several issues regarding its plantation. *C. camphora* is an exotic species in the area of sub-continent and there’s a problem with the traditional methods of propagation for instance from seeds propagation. Seeds are produced in huge amount within fruits (approximately up to 100,000 seeds per adult tree) [30] but in Pakistan production of flowers are rare and non-viable therefore seeds are not produced. Cuttings and layering are also methods that are frequently used in propagation but the limitations of resources and poor response prevent these techniques from being widely utilized at commercial level. Consequently, the conventional methods of breeding which are described above are no longer be able to satisfy the rising demand of *C. camphora* seedlings in therapeutical and pharmaceutical industries. While on the other hand, because of its versatile therapeutic properties, the annual demand of camphor is rising day by day. The global camphor market predicts that between 2020 and 2030 its demand will expand at compound annual growth rate of 11–13% (https://www.lucintel.com/camphor-market.aspx).

Plant tissue culture has been widely used for the multiplication of this important tree species because this technique has very high regenerative efficiency. Moreover, in micro-propagation, there are no limitations of climatic conditions, seasonal affect and regional restrictions, although application of this technique is very limited with regard to *C. camphora* tree. A desirable method for plant regeneration and for conservation of genetic diversity as well as use of genetic resources like bioactive substances is possible by tissue culture technique [31] For this purpose, the growth of plant is regulated by utilizing PGRs (plant growth regulators) in various concentrations and combinations while using various explants (nodal, leaves, internodal). Externally applied PGRs have potential to alter the physiology as well as internal polarity of explant. *C. camphora* can be regenerated by two pathways; somatic embryogenesis or organogenesis under controlled conditions. Based on the mentioned methods and strategies, there is a dire need to consider *in vitro* propagation strategies for this economically significant medicinal plant. Environmental conditions also effects on the phytochemical compositions of plants as well as the antioxidant capacity [32]. However, the composition of phytochemicals may vary with plant species based on the specific effect and type of plant growth regulators [33].

Because of its high antioxidant content and diverse phytochemical profile, *C. camphora* has substantial therapeutic value and is a prime target for biotechnological research. Although the bioactivity of its natural leaf extracts has been proven, but the potential of *in vitro* callus cultures as a sustainable and regulated method for increased metabolite production is not explored as much. A significant research gap exists in the comparative assessment of phytochemical and antioxidant enzyme activities between field-grown plants and callus cultures of *C. camphora*. To address this, we hypothesized that callus cultures induced under optimized *in vitro* conditions would exhibit a significantly higher phytochemical yield and enhanced antioxidant enzyme activity as compared to extracts from field-grown leaves. Therefore, the main objective of this research was to establish callus cultures via a novel protocol and conduct a comparative analysis of their phytochemical and antioxidant profiles against fresh leaf extracts. Our findings support the hypothesis that controlled *in vitro* conditions can significantly enhance the accumulation of bioactive compounds, offering a viable biotechnological platform for the mass production of valuable antioxidants from *C. camphora*.

## Materials and methods

### Plant material and culture conditions

Disease- free young nodes and fresh juvenile leaves of *Cinnamomum camphora* L. were used as an explant during this research work. These explants were procured from a healthy 20-year-old single tree of *C. camphora* grown at Quaid-e-Azam Campus, University of Punjab, Lahore, Pakistan (31.50° N, 74.30° E), in November and December 2023. Murashige and Skoog [34] (MS) medium and Woody Plant Medium (WPM) [35] with 30 gL^-1^ sucrose, 8.0 gL^-1^ agar were used with different concentrations of 2, 4-D (2, 4-dichlorophenoxyacetic acid), BAP (6-Benzylaminopurine), NAA (1-Naphthaleneacetic acid), KIN (Kinetin), IBA (Indole butyric acid), TDZ (Thidiazuron), IAA (Indole- acetic-acid), and Zeatin for this experiment on the basis of our preliminary experiments. Explants were cleaned with running tap water 4–5 times to remove all the particles of dust. They were then dipped in a solution of household detergent for 15 minutes while being continuously stirred. After rinsing with distilled water these segments were treated with 15% (*v/v*) bleach for 15 minutes with gentle agitation and washed with autoclave distilled water in laminar air flow chamber. Explants were also treated with 0.1% solution of mercuric chloride (HgCl_2_), depending on maturity of explant, with gentle agitation and washed with autoclave distilled water in laminar air flow chamber then immersed in 70% ethanol for 1 minutes and then blot-dried using sterilized blotting paper after rinsing again with autoclave distilled water. These explants were then inoculated into the test tube containing medium while keeping them near spirit lamp to avoid any contamination. These cultures were then kept under 16-hours photoperiod (40 μmol m^-2^ s^-1^) and 8 hours dark period provided by white flourescent light (36 Watt) at 25 ± 2°C temperature in the culture room.

### Data collection for morphological attributes

Data were recorded in terms of callus texture, days and frequency of calli formation, growth index and color of calli and also for the number of shoots, percentage response and number of leaves for all the tested media.

### Shoot induction and root formation in MS and WPM medium

Nodal segments were used as an explant in MS and WPM supplemented with various combinations and concentrations of plant growth regulators for shoot induction and root formation. The detail of various combinations used were as follows: M0 (MS medium without PGRs); M1 (2, 4-D 0.5 mg L^-1^ + BAP 2.0 mg L^-1^); M2 (2,4-D 0.9 mg L^-1^ + KIN 1.0 mg L^-1^); M3 (BAP 1.0 mg L^-1^ + KIN 1.0 mg L^-1^); M4 (TDZ 0.1 mg L^-1^ + 2,4-D 0.5 mg L^-1^); M5 (Zeatin 1.0 mg L^-1^ + KIN 1.0 mg L^-1^); M6 (IBA 1.0 mg L^-1^ + AC 2.0 g); M7 (IAA 0.4 mg L^-1^ + BAP 2.0 mg L^-1^) and same concentrations and combinations of PGRs were used for WPM. The media combinations were selected on the basis of preliminary experiment (data not shown here). After inoculation, the data were recorded for % response (Eq. 1), number of shoots and leaves, days for shoot initiation and leaves formation.


$$\%\text{ Response}=\frac{no.\;of\;grown\;explant}{\text{total no}.\text{ of cultured explants}}\;\times \text{1}\text{0}\text{0}$$ (Eq. 1)

### Callus initiation and its proliferation

Leaves and nodal segments were used as explants for initiation of callus in MS media supplemented with various concentrations of plant growth regulators, C0 (MS medium without PGRs); C1 (2,4-D 0.5 mg L^-1^ + BAP 2.0 mg L^-1^); C2 (Zeatin 1.0 mg L^-1^ + KIN 1.0 mg L^-1^); C3 (BAP 2.9 mg L^-1^ + KIN 1.0 mg L^-1^); C4 (TDZ 0.1 mg L^-1^ + BAP 2.0 mg L^-1^); C5 (IBA 0.5 mg L^-1^ + NAA 0.5 mg L^-1^) used for callus induction from leaf while M0 (MS medium without PGRs); M1 (2, 4-D 0.5 mg L^-1^ + BAP 2.0 mg L^-1^); M2 (2,4-D 0.9 mg L^-1^ + KIN 1.0 mg L^-1^); M3 (BAP 1.01.0 mg L^-1^ + KIN 1.0 mg L^-1^); M4 (Zeatin 1.0 mg L^-1^ + KIN 1.0 mg L^-1^); M5 (IBA 0.5 mg L^-1^ + BAP 1.0 mg L^-1^). After 30 days of inoculation the data were recorded for % frequency of callus formation (Eq 2), Growth index (Eq. 3), days for callus initiation, callus texture and color.


$$Frequency\;of\;Callus\;formation\;(FCF)\%=\frac{no.\;of\;Callus\;initiation}{\text{total no}.\text{ of cultured explants}}\;\times \text{1}\text{0}\text{0}$$ (Eq. 2)

The fresh weight of callus formation/growth index was measured using following formula:


$$Growth\;Index\;(GI)=\frac{Final\;weight\;(Wf)-Initial\;Weight(Wi)}{Initial\;Weight(Wf)}$$ (Eq. 3)

Wi= initial weight.

Wf= final weight.

### Sub-culturing of Calli for their Proliferation on MS medium supplemented with various combinations of Plant Growth Regulators

In vitro grown calli were divided into small segments by excising the calli established on basal medium (MS medium) supplemented with different growth regulators. These calli were shifted on fresh MS medium containing various growth regulators, designated as S1(0.5 mg L^-1^ 2,4-D + 2 mg L^-1^BAP), S2 (0.5 mg L^-1^ 2,4-D + 0.1 mg L^-1^TDZ) and S3 (1.7 mg L^-1^ IAA + 2 mg L^-1^ BAP) with the interval of 20 days to maintain healthy calli. After shifted calli on media, they were placed under white fluorescent light (36 Watt) at 25 ± 2°C temperature. Before exposing to light [(14-hour light) (36 Watt)], they were maintained in the dark for five days at 25 ± 1°C temperature in the culture room. After one month, calli were well-established and they were further used for various biochemical analysis.

### Comparative Analysis of antioxidant enzyme activities and phytochemicals of leaf extract and callus culture of *C. camphora* L

#### Extraction and estimation of antioxidant enzymes.

Healthy and fresh leaves of *C. camphora* were collected and packed in polythene bags and brought to Plant Developmental and Regenerative Biology Laboratory, Institute of Botany, University of the Punjab, Lahore. This plant material was rinsed under running tap water thoroughly and dried with the help of tissue paper by gentle tapping. Afterwards, leaves (4g) were placed in −80°C for 24 hrs. Similarly, 4 g calli were used for the extraction of enzymes. Plant material and calli were crushed with the help of pestle and mortar separately. Phosphate buffer (7.2 pH) was used with 1:2 of crude extract and 0.1g PVP (*Polyvinyl polypyrrolidone*; Sigma Aldrich) was added to make homogenous slurry. Slurry was centrifuged at 4°C and 14000 rpm for 15 minutes. After centrifugation, supernatant was used for estimation of antioxidant enzyme activities and phytochemical analysis. Extraction percentage was calculated by using following formula (Eq. 4):


$$Extraction\;percentage(\%)=\frac{\text{W1}}{\text{W2}}\times \text{100}$$ (Eq. 4)

W1 = weight of crude extract

W2 = weight of sample

#### Estimation of peroxidase (E.C 1.11.1.7) activity (POD).

The ‘Guaiacol-H_2_O_2_’ method described by Luck [36] was modified for quantitative estimation of peroxidases. Two test tubes were used, one with a reaction mixture; 3.0 mL of phosphate buffer (0.1M; pH 7.2) with 0.05 mL of guaiacol (20 mM; 2-methoxyphenol) and 0.1 mL of crude enzyme extract. Instead of crude enzyme extract, 0.1 mL of distilled water was used in the second test tube. In both test tubes, 0.03 mL H_2_O_2_ (12.3 mM) was added for the reaction. Peroxidase activity was estimated as the time required for increase in absorbance of 0.1 (0.4–0.5) at 240 nm and represented as UmL^-1^ of enzyme (Eq. 5).


$$Enzyme\;activity=(\frac{\text{500}}{\Delta }t)\times (\frac{\text{1}}{\text{1000}})\times (\frac{TV}{\text{UV}})\times (\frac{\text{1}}{fwt})$$ (Eq. 5)

Δt = change in time; TV = total prepared volume (mL); VU = volume of the sample used (mL); fwt = fresh weight (g).

#### Estimation of catalase (E.C 1.11.1.6) activity.

The activity of catalase was measured using the method described by Beers and Sizer [37], with some modifications. The reaction was conducted with two buffer solutions (A and B). Reagent A contained 50 mM potassium phosphate at pH 7.0 while B was 0.036% H_2_O_2_ solution with 50 mM potassium phosphate buffer. The reaction mixture of catalase was composed of 0.1 mL of enzyme extract and 2.9 mL of Reagent B, while the blank contained only 3.0 mL of Reagent A. The enzyme activity was determined by measuring the required time for decrease in absorbance from 0.45 to 0.40 (at 240 nm) and was expressed as UmL^-1^ of enzyme (Eq. 6).


$$Catalase\;activity\;(\frac{units}{ml}enzyme)=\frac{\text{3}.\text{45}\times \text{df}}{\text{Min}\times \text{0}.\text{1}}$$ (Eq. 6)

3.45 = Decomposition of 3.45 micromoles of hydrogen peroxide in a 3.0 ml. of reaction mixture that produce decrease in the A_240nm_ from 0.45 to 0.40 units.

df = dilution factor (mL); Min = Time in minutes required for the A_240nm_ to decrease from 0.45 to 0.40 absorbance units; 0.1 = Volume of enzyme used (mL).

#### Estimation of superoxide dismutase (E.C 1.15.1.1) Activity (SOD).

The activity of superoxide dismutase was measured by using a modified method developed by Maral and co-researchers [38]. Reaction mixture was prepared by adding 1 mL of sodium cyanide (NaCN), 10 ml of Methionine, 10 mL of EDTA, 1mL of NBT (Nitroblue tetrazolium), and 1 mL of Riboflavin as a substrate. The volume of the mixture was made up to 100 mL by adding buffer solution. Two test tubes (15 × 150 mm) were used for the assay; one contained 5 µL of extract and reaction mixture, while the other tube served as a blank, contained 2 mL of reaction mixture only. Both the tubes were placed under fluorescent bulbs (30 Watt) for 15 minutes. By using spectrophotometer (UV- 4000S), the absorbance of both test tubes was measured at 560 nm. SOD activity was further calculated on the basis of this fact that 50% inhibition is caused by one unit of SOD. SOD activity was expressed as units mg^-1^ of protein. The % inhibition of NBT was calculated to determine SOD as follows (Eq. 7):


$$\%\;inhibition=\frac{\text{Absorbance of control sample}-\text{Absorbance of experimental sample}}{\text{Absorbance of experimental}}\;\times \text{100 }$$ (Eq. 7)

#### Estimation of glutathione peroxidase (E.C 1.11.1.9) activity (GPX).

To measure the activity of GSH-Px, the method of Flohé and Günzler [39], was slightly modified by using H_2_O_2_ (Duksan reagent) as a substrate. To conduct the enzyme reaction, 200 µL of the supernatant was mixed with 400 µL of 0.1mM GSH (reduced glutathione; Sigma) and 200 µL of Na_2_HPO_4_ (0.067 M) in a test tube. For the non-enzyme reaction, the same reagents were used without the supernatant. The mixture was pre-heated on a water bath at 25°C temperature for 5 minutes, followed by the addition of 200 µL of 1.3 mM H_2_O_2_ to initiate the reaction. This reaction was lasted for 10 minutes and then stopped by adding 1 mL of 1% trichloric acetic acid (TCA; Merck) and by placing the mixture into an ice bath for 30 minutes. The supernatant was collected by centrifuging the mixture for 10 minutes at 3000 rpm, 480 µL of the supernatant was added into a test tube with 2.2 mL of Na_2_HPO_4_ (0.32 M) and 0.32 mL of 1.0 mM 5,5-dithio-bis (2-nitrobenzoic acid; DTNB) was added for development of color. The absorbance was measured at 412 nm with a spectrophotometer within 5 minutes. GPX activity is expressed as Umg^-1^. Residual glutathione concentration was calculated by using standard curve of the glutathione. GPX activity is equals to the no. of µmol consumed glutathione (Eq. 8).


$$Glutathione\;peroxidase\;activity\;\left(\frac{units}{mg}\right)=\frac{Conc.\;of\;GSH\;in\;STD-Conc.\;of\;residual}{time\;(\text{1}\text{0}\;mintues)}\times \frac{Total\;volume(ml)}{volume\;of\;sample(ml)}\times \text{D}.\text{f}$$ (Eq. 8)

One unit of GPX is defined as the amount of enzyme that has capability to oxidize 1.0 µM GSH to GSSG per minute at temperature 25°C.

#### Estimation of total phenolic contents.

Chun and Kim [40] method was used to determine the total phenolic content involved mixing of 0.2 mL of the extract with 2.6 mL of distilled water and adding the 0.2 mL of Folin-Ciocalteu reagent after 5 minutes, then 2 ml of 7% Na_2_CO_3_ was added and stirring for 30 seconds. This solution left in the dark for almost 90 minutes and then absorbance was measured at 750 nm against blank (without enzyme extract). The amount of total phenolic contents was measured in mg GAE g^-1^. This measurement was obtained by using a standard curve of Gallic acid calibration at 750 nm (Eq. 9).


$$\text{TPC}=c\frac{V}{m}$$ (Eq. 9)

c = concentration of gallic acid obtained from calibration curve in mg/mL

v = volume of extract in mL; m = mass of extract in gram

### Determination of total flavonoid contents

To measure the amount of flavonoids, Ivanova [41] method was employed that involved using the AICI_3_-NaNO_2_-NaOH complex. The test tube contained 0.2 mL of the extract with 3.5 mL of distilled water, 0.15 mL of 5% NaNO_2_, 0.15 mL of 10% AlCl_3_, and 1 mL of 1 M NaOH, with the interval of 5 minutes. The reaction mixture was left to react for 15 minutes, and the absorbance was measured at 510 nm. Flavonoid content was reported in mg CatE g^−1^, which was calculated from the standard curve of catechin calibration at 510 nm (Eq. 10).


$$\text{TFC}=c\frac{V}{m}$$ (Eq. 10)

c = concentration of catechin obtained from calibration curve in mg/mL

v = volume of extract in mL; m = weight of plant extract in g

### Experimental layout and statistical analysis

All experiment was performed with 10 replicates for each treatment of calli induction and shoot formation. For the analysis of phytochemical contents and antioxidant activity 3 readings were recorded for each sample of all treatments. By using statistical program SPSS (Version 25.0.0), all the recorded data were analyzed to find out means and standard errors conducting ANOVA. LSD and Duncan’s Multiple Range test was also used to find statistically significant differences among the means of various treatments. Differences were considered as significant at *P* ≤ *0.05*.

## Results

### Standardization of the medium for shoot initiation of *C. camphora*

Young juvenile explant of *C. camphora* was collected during the months of November and December 2023. Nodal sections were inoculated on MS media containing various combinations of growth regulators. *In vitro* plants were raised to get healthy germplasm under the controlled environmental conditions. Explant treated with 10% bleach gave best surface sterilization of explant as compared to other sterilant used during this investigation and only 20% contamination was observed. For *in vitro* establishment of plant only nodal sections were used as explant during this investigation. After reviewing literature, two types of media, MS and WPM with various growth regulators were tried alone and in combination with PGRs however, only MS media fortified with different concentrations of PGPRs were proved effective. Shoot formation and callus induction were not observed on WPM media. So, here we presented the results regarding to MS media only.

### Shoot initiation of *C. camphora* in MS medium supplemented with different plant growth regulators

**Fig 1:**
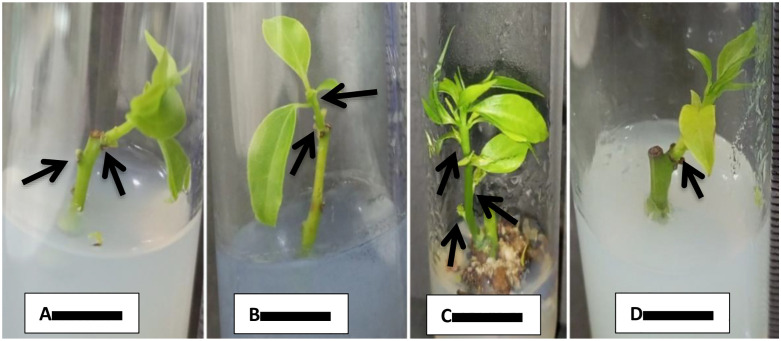
A: Shoot initiation from nodal segment on MS medium fortified with BAP and IAA (2.0 mg L^-1^ +0.4 mg L^-1^) after 22 days. B: On MS basal media fortified with IBA (1.0 mg L^-1^) and AC 2.0g after 20 days. C: On MS basal media fortified with BAP and 2, 4-D (2.0 mg L^-1^+ 0.5 mg L^-1^ mg L^-1^) after 30 days. D: On MS basal media fortified with TDZ and 2, 4-D (0.1mg L^-1^ + 0.5 mg L^-1^) after 22 days. Bar = 1 cm.

For shoot initiation of *C. camphora,* MS media were used with various combinations of PGRs and activated charcoal (2.0 gL^-1^) was also used for reducing hyperhydricity (vitrification). Eight media were used for shoot initiation from nodal segment. Best results were observed on M1 medium with 100% response (Fig 1C), although it took more time (24.67 days) to initiate shooting after inoculation but it produced more number of leaves (11.00) and shoots (1.33) as compared to other tested media. Similarly, M4 medium also showed 100% response within 18.00 days but number of leaves (6.33) and number of shoots (1) were less (Fig 1D) as compared to M1. Shoot initiation response was also 100% on M6 media within 19 days of inoculation. Here, the number of leaves (5.66) and number of shoots (1) were also less as compared to M1 medium (Fig 1B). M7 showed 60% response after 14.33 days of inoculation and produced 4.33 leaves and 0.67 shoots (Fig 1A). During this investigation, M0, M2, M3, and M5 media were not responded regarding shoot formation (Table 1).

**Table 1:** Shoot initiation from nodal segment of *C. camphora* on MS medium supplemented with various concentrations of plant growth regulators.

Medium MS	Treatments	Days for Shoot Initiation	% Response	No. of Leaves	No. of shoots
**M0**	0	0	0	0	0
**M1**	2, 4-D (0.5 mg L^-1^) + BAP (2.0 mg L^-1^)	24.67 ± 1.76^a^	100	11.00 ± 2.64^a^	1.33 ± 0.33^a^
**M2**	2, 4-D (0.9 mg L^-1^) +KIN (1.0 mg L^-1^)	0	0	0	0
**M3**	BAP (1.0 mg L^-1^) + KIN (1.0 mg L^-1^)	0	0	0	0
**M4**	TDZ (0.1 mg L^-1^) + 2, 4-D (2.26 mg L^-1^)	18.00 ± 1.52^ab^	100	6.33 ± 1.45^b^	1.00 ± 0.00^ab^
**M5**	Zeatin (1.0 mg L^-1^) + KIN (1.0 mg L^-1^)	0	0	0	0
**M6**	IBA (1.0 mg L^-1^) + AC (2.0 g)	19.00 ± 0.57^ab^	100	5.66 ± 0.88^b^	1.00 ± 0.00^ab^
**M7**	IAA (0.4 mg L^-1^) +BAP (2.0 mg L^-1^)	14.33 ± 7.17^b^	60	4.33 ± 2.96^bc^	0.67 ± 0.33^b^

Values are the mean ± S.E of ten replicates for each treatment.

Different alphabetical letters in each column shows the significant differences at P ≤ 0.05 according to Duncan’s multiple range test.

### Standardization of Medium for Callus Induction and Maintenance of *C. camphora*

MS media in combination with various growth regulators were tried for callus induction and proliferation of *C. camphora.* Seven media were used for callus induction from leaf explants as mentioned in Table 2. From these media best responded medium was used for further proliferation of callus.

**Table 2:** Callus induction from leaf explant of *C. camphora* on MS medium fortified various combinations of plant growth regulators.

Medium	Treatments	Days to callus induction	FCF %	Morphology
**C0**	0	0	0	
**C1**	2,4-D (0.5 mg L^-1^) +BAP (2.0 mg L^-1^)	26.2 ± 1.46^b^	100	Brownish in color with dark patches, friable and compact
**C2**	Zeatin (1.0 mg L^-1^) + KIN (1.0 mg L^-1^)	0	0	
**C3**	BAP (2.9 mg L^-1^) +KIN (1.0 mg L^-1^)	0	0	
**C4**	TDZ (0.1 mg L^-1^) +BAP (2.0 mg L^-1^)	57.2 ± 1.39^a^	100	Soft callus, Whitish Yellow, friable
**C5**	IBA (0.5 mg L^-1^) +NAA (0.5 mg L^-1^)	0	0	
**C6**	IAA (1.7 mg L^-1^) +BAP (2.0 mg L^-1^)	0	0	

Values are the mean ± S.E of ten replicates for each treatment.

Different alphabetical letters in each column shows the significant differences at P ≤ 0.05 according to Duncan’s multiple range test.

### Effect of different combinations of plant growth regulators on the leaf explant for callus induction

**Fig 2:**
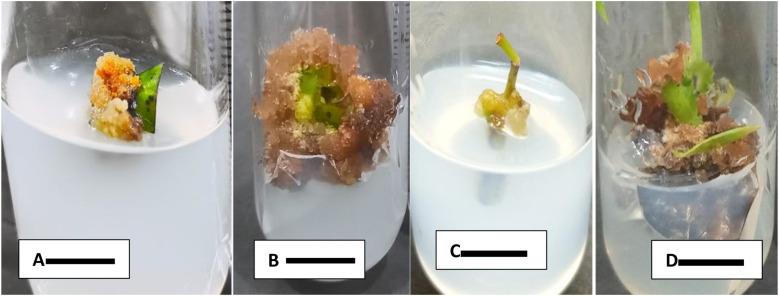
A: Callus induction from leaf on MS medium with TDZ and BAP (0.1 mg L^-1^+ 2.0 mg L^-1^) after 57 days. B: On MS medium with BAP and 2, 4-D (2.0 mg L^-1^ + 0.5mg L^-1^) C: MS medium supplemented with BAP and IBA (1.01.0 mg L^-1^+ 0.5 mg L^-1^) after 22 days. D: Callus induction in MS medium supplemented with BAP and 2, 4-D (2.0 mg L^-1^+ 0.5 mg L^-1^) after 30 days. Bar = 1 cm.

From all the seven media only two were proved effective for callus induction with 100% response. C1 medium showed best results as compared to C4 with regards to days for callus induction. In case of C1 medium, calli were induced within 26.2 days after inoculation (Fig 2B). Morphologically, callus was brown in color with dark patches on it, and friable in texture. Callus was induced after 57.2 days on C4 culture medium (Fig 2A). Callus produced on this medium was soft, whitish yellow in appearance and friable in texture. The entire calluses were produced from the leaf discs on both C1 and C4 medium were non-embryogenic.

### Effect of different combinations of plant growth regulators on nodal segment for callus induction and maintenance of *C. camphora*

Six media combinations were used for callus induction from nodal explants, calli were induced in two (M5 and M1) combinations, M1 media showed 100% callus induction after 22.70 days of inoculation. Calli derived from M1 medium were brownish yellow and leathery in texture after 22 days of inoculation, but after maintaining, it turned into dark brown and friable texture (Fig 2D). It was observed that the hard calli produced from nodal segment in the presence of 2, 4-D and BAP were non-embryogenic with no regenerative ability. Callus Initiation was supported about 60% in M5 media within 17.40 days but the rate of callus induction was slowest as compared to M1. Calli derived from M5 were also non-embryogenic. The deceleration in growth was observed after 50^th^ days of inoculation. However, on M5 medium calli were creamy yellowish and leathery in texture (Table 3)

**Table 3:** Callus initiation from stem segment in MS medium supplemented with plant growth regulators.

Medium MS	Treatments	Days to callus initiation	% Response	Callus morphology
**M0**	0	0	0	0
**M1**	2, 4 D (0.5 mg L^-1^)+BAP (2.0 mg L^-1^)	22.70 ± 0.50^a^	100	Hard callus, brownish yellow and compact in texture
**M2**	2,4-D (0.9 mg L^-1^) +KIN (1.0 mg L^-1^)	0	0	
**M3**	BAP (1.0 mg L^-1^) + KIN (1.0 mg L^-1^)	0	0	
**M4**	Zeatin (1.0 mg L^-1^)+ KIN (1.0 mg L^-1^)	0	0	
**M5**	IBA (0.5 mg L^-1^) +BAP (1.0 mg L^-1^)	17.40 ± 7.11^b^	60	Soft callus, creamy yellowish in color and leathery in texture

Values are the mean ± S.E of ten replicates for each treatment.

Different alphabetical letters in each column shows the significant differences at P ≤ 0.05 according to Duncan’s multiple range test.

### Sub-culturing of callus for maintenance and proliferation

**Fig 3:**
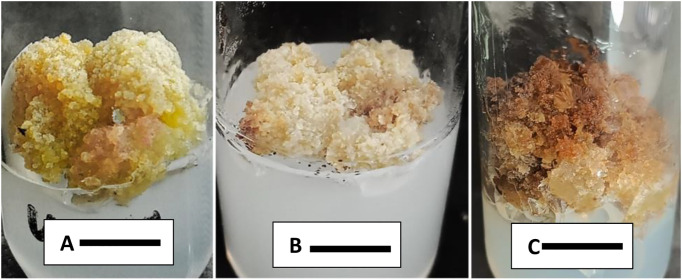
A: Callus proliferation, in response of sub-culturing on S1 [(MS medium with BAP + 2, 4-D) (2.0 mg L^-1^+ 0.5 mg L^-1^)] after 20 days. B: On S3 MS medium with IAA + BAP (1.7 mg L^-1^ + 2.0 mg L^-1^) after 20 days. C: On S2 MS medium with TDZ+ 2,4-D (0.1 mg L^-1^+ 0.5 mg L^-1^ mg L^-1^) after 20 days. Bar= 1cm.

Successful sub-culturing of callus produced from nodal segment/leaf explant was done on three media; S1, S2 and S3. Combinations and concentrations of S1 medium was same as used before for callus induction from nodal segment while two new media combination (S2 and S3) were used for further proliferation of callus cultures. S1 medium supported the production of healthy calli within 19.20 days. Morphologically, calli were soft and lush green in color, lightly friable, granular and compact in texture. Callus proliferation on S2 and S3 media took 18.40 days after inoculation (Table 4). Calli in S2 media were reddish brown in color with creamy base, lightly compact and friable in texture. On S3 media calli were whitish in color with light brown patches on it and granular in texture. The highest growth index (0.76 g) was recorded on S1 media as compared to S2 with 0.47 g and S3 with 0.66 g. These proliferated calli (0.5g) were used for analysis of antioxidant enzyme activity and phytochemicals (Figs 3).

### Comparative study of antioxidant enzymes and phytochemicals of *C. camphora* leaf extract and callus cultures

#### POD, CAT, SOD and GPX activity.

**Fig 4:**
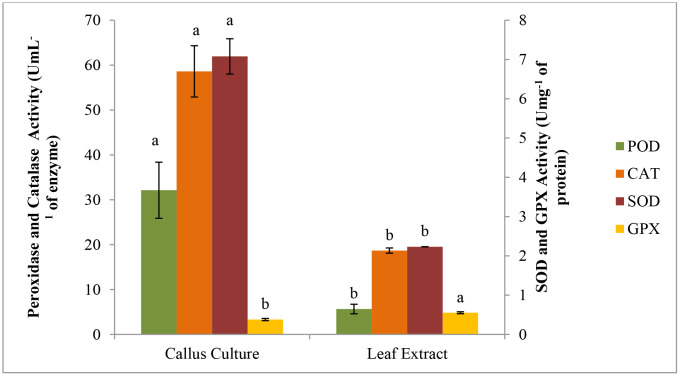
A comparison of antioxidant enzymes activity of of callus culture and leaf extract of *C. camphora.*

Overall, POD activity of callus culture was found higher as compared to leaf extract of *C. camphora*. A Decreasing trend in the activity of peroxidases was recorded from 32.124 to 5.66 UmL^-1^ of enzyme in callus as compared to leaf extract. Similar trend was also recorded in the activity of catalases, leaf extract showed 18.683 as compared to 58.6 UmL^-1^ of enzyme activity in callus culture. SOD activity of callus cultures was 7.078 Umg^-1^ of protein while in case of leaf extract 2.232 Umg^-1^ of protein was recorded. Unlike the above mentioned three primary antioxidants, GPX activity of leaf extract was higher as compared to callus cultures grown under the controlled conditions. Decreasing trend in the activity of GPX was from 0.552 to 0.379 Umg^-1^ of protein (Fig 4).

### Total phenolic and flavonoid contents

**Fig 5:**
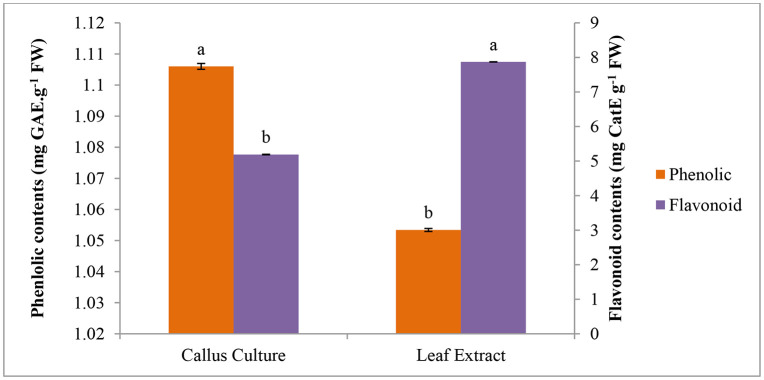
A comparison of phenolic and flavonoid contents of callus culture and leaf extract *C. camphora.*

The phenolic contents in callus culture were 1.106 mg GAE g^-1^ and in leaf extract were 1.053 mg GAE g^-1^. Overall, callus culture showed highest phenolic contents as compared to leaf extract. A decreasing trend in flavonoid contents was recorded from 7.87 to 5.189 mg CatE g^-1^. Leaf extract of plant showed highest flavonoid contents (7.87 mg CatE g^-1^) while in callus cultures flavonoid contents were 5.189 mg CatE g^-1^ (Fig 5).

## Discussion

In present study, two type of plant material (young leaves and nodal segments) were used as explant for shoot induction of *C. camphora*. Shoot initiation from nodal segments was achieved for establishment of *in vitro* plants. Different combinations of PGRs in MS media were tried for shoot induction. MS medium containing 0.5 mg L^-1^ 2, 4-D + 2.0 mg L^-1^ BAP provided best results with lateral shoot induction after 22 days as compared to other tested media. Similar results were recorded by Du [42] who suggested that same combination and concentration of 2, 4-D and BAP for direct organogenesis by nodal explant from seedlings of *C. Camphora.* Somatic embryogenesis in cell suspensions of *Cenchrus ciliaris* L. on the similar combination of PGRs was achieved by Goyal [43]. It is worth mentioning here that 2, 4-D and BAP were prove more effective in triggering the cell elongation as compared to other growth regulators [44,45]. When the combination of 0.1 mg L^-1^ TDZ and 2.26 mg L^-1^2, 4-D was used, shoots developed within 18 days without lateral shoot formation. Pakum [46] demonstrated similar results within 8 weeks for direct organogenesis from leaf explant of *Kalanchoe tomentosa* Baker (Panda plant) on 0.1 mg L^-1^ TDZ and 0.5 mg L^-1^ 2, 4-D separately while Li [47] investigated callus induction from leaf explant of *Bupleurum chinense* on 2 mg L^-1^ 2, 4-D and 1 mg L^-1^ TDZ. Similarly, application of only TDZ in MS medium was also documented as effective for *C. camphora* [48]. Lower level of TDZ (cytokinin) plays an important role in shoot induction and accelerates the plant regeneration [49]. BAP in various combinations was also previously used for shoot initiation of *C. camphora* explants [50]. During this investigation, MS medium with 1.0 µM IBA + 2.0 g L^-1^ AC (activated charcol) was also showed 100% response of shoot initiation however, these results were contradictory to Babu [51], who reported rooting and shooting on WPM medium with similar combination of medium. The above-mentioned studies and our results confirmed that higher concentration of cytokinin’s with various concentrations of auxin supports the shoot induction.

In this investigation, the response of callus induction from young juvenile leaf and the nodal segments was also evaluated and found that significant and more positive response was observed on MS medium with combination of 2.0 mg L^-**1**^ BAP and 0.5 mg L^-1^2,4-D as compared to other tried media. Similar results for callus initiation and proliferation from leaf disc of *C. camphora* were described by Chen [52] but in their case calli were induced on nodal explants instead of leaf segments. However, in another study, various combinations of auxins and cytokinin’s were describes for callus initiation from leaf explants of *C. camphora* [50,53]. Similarly, the combination of 2,4-D and BAP was also reported as good for callus induction and proliferation in many other plant species [54–56]. Since, combination of 2,4-D and BAP synergistically more effective in promoting callus development by triggering the cell elongation [57–59]. 2, 4-D alone was also suggested as the best auxin for callus induction and proliferation in many previous studies [60–62]. In present study, the morphology of developed callus was lush green while compact in texture. Muthi’ah [55] observed similar morphology of callus on this combination of growth regulators in his study on *Calotropis gigantea*. Aref and Salem [53] found different morphology of calli of *C. camphora* on different combinations of PGRs by using different explants. According to Abu-Romman [63] and Yaroshko [64], even within the same genus, the appearance of callus depends upon the concentration and type of plant growth regulators. In present investigation, maximum proliferation response, growth index of callus (0.76g within 19 days) after callus sub-culturing was recorded at 2.0 mg L^-**1**^ BAP + 0.5 mg L^-1^ 2, 4-D. Morphologically, callus was lush green, granular, lightly friable and compact in nature. El-Kader [65] reported similar results by sub-culturing of *C. camphora* leaf callus on MS medium fortified with 2.0 mg L^-1^ NAA and 1.0 mg L^-1^ BAP. However, contrary to our results, best yield of 11 g was obtained after 45 days of sub-culturing in their study. For high yield of callus various combinations of auxins and cytokinin’s were used in literature [66,67]. Time interval of callus sub-culture varies among various species in literature also [68].

In current study, a comparative analysis of phytochemicals and antioxidant activities was assayed in callus vs. leaf extract of *C. camphora*. The proliferated callus cultures were used for comparative analysis of phytochemical and antioxidant activity. Junairiah [69] and Astuti [70] reported that compact calli were far better regarding to production of secondary metabolites as compared friable ones. Callus culture showed great potential to produce secondary metabolites, i.e., phytochemicals and antioxidants those were also found in whole plants. Various investigations and approaches have been done for the production and to overcome the limitations of these vital secondary metabolites. For different applications of biotechnological fields these *in vitro* extract of callus cultures were utilized successfully [71,72]. In our findings, an increasing trend in the activities of peroxidase, catalase, and superoxide dismutase was observed in callus culture as compared to leaf extract . These results are in line with the previous study of Ali [73] who demonstrated that the application of GA_3_ (PGR) on *sorghum* seedlings resulted in increasing SOD and POD activity. POD, SOD and catalase were considered as important enzymes which activate the defense mechanisms of plant by regulating metabolic processes [74,75]. Reason behind this increasing trend might be the inhibition of reactive oxygen species (ROS) by a variety of phytochemicals and antioxidant enzymes [76], which are essential for protecting the calli cells from various stress factors. In present study, a decreasing trend was found in Glutathione peroxidase activity. In plant, GPXs may play a variety of functions in stress tolerance and growth [77]. It was reported that GPX activity results in accumulation of high rate of ROS in zygotic or embryonic nuclei [78] and thought to have signaling factors during abiotic stress [79]. This study was in line with Passaia [80] and Madhu [81] who described that GPXs control the plant growth and development in both favorable and unfavorable environments. SOD and GPx have the ability to directly balance the oxidative stress and provide protection to plant cells from DNA damage [82]. SOD initially, catalases the dismutation of molecular oxygen (O_2_) and released hydrogen peroxide (H_2_O_2_) while the primary enzyme that breaks down this hydrogen peroxide into water in cells is glutathione peroxidase [83].

**Table 4:** Callus Proliferation after Sub-culturing on MS medium supplemented with various combinations of Plant Growth Regulators.

Medium	Treatments	Days to callus growth	Growth index (g)	Morphology
S1	2,4-D (0.5 mg L^-1^) + BAP (2.0 mg L^-1^)	19.20 ± 1.39^a^	0.76 ± 0.06^a^	Soft callus, lush green in color, lightly friable, granular and compact in texture
S2	2,4-D (0.5 mg L^-1^) +TDZ (0.1 mg L^-1^)	18.40 ± 1.63^a^	0.47 ± 0.13^a^	Reddish brown with creamy base, light compact and friable
S3	IAA (1.7 mg L^-1^) + BAP (2.0 mg L^-1^)	18.40 ± 0.58^a^	0.66 ± 0.10^a^	Whitish in color with light brown patches, granular in texture

Values are the mean ± S.E of ten replicates for each treatment.

Different alphabetical letters in each column shows the significant differences at P ≤ 0.05 according to Duncan’s multiple range test.

In present work, statistically significant increase in phenolic contents and decrease in flavonoid contents was recorded in callus cultures as compared to leaf extract grown in natural conditions. Medicinal plants with high concentrations of phenols and flavonoids have strong antioxidant effects. Our findings indicated that the phenylpropanoid pathway is activated by PGRs-containing media because this pathway causes an increase in synthesis of phenolic contents [84]. It is reported that Phenylalanine ammonia-lyase (PAL) enzyme activity increased by application of plant growth regulators exogenously and it causes the accumulation of phenols and other secondary metabolites [85,86]. As Rybin [56] has been reported the combination of 2,4-D and BAP for active production of phenolics and callus cultures of *Vaccinium corymbosum* L. Therefore, present study revealed that phenylpropanoid molecule production was positively impacted by various concentrations of PGPRs and phenolic contents were produced in response of defense mechanism. Correlation between total phenolic content and antioxidants in other plants has been recorded [87]. According to Hatami [88], plant metabolism is quite complicated and depends on variety of variables, including the types and concentrations of growth hormones in the culture media, age, and type of cells or tissues. Overall results demonstrate that an increase in antioxidant activity and phenolic contents was due to various factors that might be the oxidative stress on *in vitro* callus cultures or different combinations of PGRs.

## Conclusion

In conclusion, it was observed during this investigation that callus cultures extract contains higher phytochemicals and antioxidant enzymes as compared to leaf extract of *C. camphora* and this enhanced activity of enzymatic antioxidants and phenolic contents might be correlated with *in vitro* conditions and PGRs used during callus induction. The results hint the potential use of callus cultures of *C. camphora* for the production of phytochemicals and antioxidants enzymes at commercial scale. These plant-based antioxidants and phytochemicals can be used in pharmaceutical, cosmetics and food industries to meet the country demand. However, this study requires more investigations to assess the efficiency of various combinations of plant growth regulators or some other elicitors to enhance these phytochemicals and enzymes under *in vitro* conditions.

## Supporting information

S1 File Data sheet. (XLSX)

## References

[1] Forni C, Facchiano F, Bartoli M, Pieretti S, Facchiano A, D’Arcangelo D, et al. Beneficial Role of Phytochemicals on Oxidative Stress and Age-Related Diseases. Biomed Res Int. 2019:8748253. doi: 10.1155/2019/8748253 31080832 PMC6475554

[2] Forni C, Frattarelli A, Lentini A, Beninati S, Lucioli S, Caboni E. Assessment of the antiproliferative activity on murine melanoma cells of extracts from elicited cell suspensions of strawberry, strawberry tree, blackberry and red raspberry. Plant Biosystems - An International Journal Dealing with all Aspects of Plant Biology. 2015;150(6):1233–9. doi: 10.1080/11263504.2015.1018981

[3] Lucioli S, Di Bari C, Nota P, Frattarelli A, Forni C, Caboni E. Methyl jasmonate promotes anthocyanins’ production in Prunus salicina × Prunus persica in vitro shoot cultures. Plant Biosystems - An International Journal Dealing with all Aspects of Plant Biology. 2016;151(5):788–91. doi: 10.1080/11263504.2016.1255267

[4] Vargas-Mendoza N, Morales-González Á, Madrigal-Santillán EO, Angeles-Valencia M, Anguiano-Robledo L, González-López LL, et al. Phytochemicals and modulation of exercise-induced oxidative stress: a novel overview of antioxidants. Am J Transl Res. 2022;14(11):8292–314. 36505319 PMC9730074

[5] Ogwu MC, Izah SC, Joshua MT. Ecological and environmental determinants of phytochemical variability in forest trees. Phytochem Rev. 2025. doi: 10.1007/s11101-025-10066-0

[6] Aryal S, Baniya MK, Danekhu K, Kunwar P, Gurung R, Koirala N. Total Phenolic Content, Flavonoid Content and Antioxidant Potential of Wild Vegetables from Western Nepal. Plants (Basel). 2019;8(4):96. doi: 10.3390/plants8040096 30978964 PMC6524357

[7] Cory H, Passarelli S, Szeto J, Tamez M, Mattei J. The Role of Polyphenols in Human Health and Food Systems: A Mini-Review. Front Nutr. 2018;5:87. doi: 10.3389/fnut.2018.00087 30298133 PMC6160559

[8] Kumar A, P N, Kumar M, Jose A, Tomer V, Oz E, et al. Major Phytochemicals: Recent Advances in Health Benefits and Extraction Method. Molecules. 2023;28(2):887. doi: 10.3390/molecules28020887 36677944 PMC9862941

[9] Siddiqui SA, Khan S, Mehdizadeh M, Bahmid NA, Adli DN, Walker TR, et al. Phytochemicals and bioactive constituents in food packaging - A systematic review. Heliyon. 2023;9(11):e21196. doi: 10.1016/j.heliyon.2023.e21196 37954257 PMC10632435

[10] Chowdhary V, Alooparampil S, V. Pandya R, G. Tank J. Physiological Function of Phenolic Compounds in Plant Defense System. Biochemistry. IntechOpen. 2022. doi: 10.5772/intechopen.101131

[11] Rácz A, Czégény G, Csepregi K, Hideg É. Ultraviolet-B acclimation is supported by functionally heterogeneous phenolic peroxidases. Sci Rep. 2020;10(1):16303. doi: 10.1038/s41598-020-73548-5 33004945 PMC7530754

[12] Merecz-Sadowska A, Sitarek P, Kucharska E, Kowalczyk T, Zajdel K, Cegliński T, et al. Antioxidant Properties of Plant-Derived Phenolic Compounds and Their Effect on Skin Fibroblast Cells. Antioxidants (Basel). 2021;10(5):726. doi: 10.3390/antiox10050726 34063059 PMC8147979

[13] Vignesh A, Selvakumar S, Vasanth K. Comparative LC-MS analysis of bioactive compounds, antioxidants and antibacterial activity from leaf and callus extracts of *\\j-fs04\J-PLOS-L\Production\PONE\pone.0321155\FROM_CLIENT\Accepted_manuscripts\Suppl\Data Sheet.xlsx*. Phytomedicine Plus. 2022;2(1):100167. doi: 10.1016/j.phyplu.2021.100167

[14] Donadio G, Mensitieri F, Santoro V, Parisi V, Bellone ML, De Tommasi N, et al. Interactions with Microbial Proteins Driving the Antibacterial Activity of Flavonoids. Pharmaceutics. 2021;13(5):660. doi: 10.3390/pharmaceutics13050660 34062983 PMC8147964

[15] Roy A, Khan A, Ahmad I, Alghamdi S, Rajab BS, Babalghith AO, et al. Flavonoids a Bioactive Compound from Medicinal Plants and Its Therapeutic Applications. Biomed Res Int. 2022;2022:5445291. doi: 10.1155/2022/5445291 35707379 PMC9192232

[16] Tajammal A, Siddiqa A, Irfan A, Azam M, Hafeez H, Munawar MA, et al. Antioxidant, molecular docking and computational investigation of new flavonoids. Journal of Molecular Structure. 2022;1254:132189. doi: 10.1016/j.molstruc.2021.132189

[17] Corso M, Perreau F, Mouille G, Lepiniec L. Specialized phenolic compounds in seeds: structures, functions, and regulations. Plant Sci. 2020;296:110471. doi: 10.1016/j.plantsci.2020.110471 32540001

[18] Tian Z, Luo Q, Li Y, Zuo Z. Terpinene and β-pinene acting as signaling molecules to improve Cinnamomum camphora thermotolerance. Industrial Crops and Products. 2020;154:112641. doi: 10.1016/j.indcrop.2020.112641

[19] Zhang G, Yan X, Wu S, Ma M, Yu P, Gong D, et al. Ethanol extracts from Cinnamomum camphora seed kernel: Potential bioactivities as affected by alkaline hydrolysis and simulated gastrointestinal digestion. Food Res Int. 2020;137:109363. doi: 10.1016/j.foodres.2020.109363 33233066

[20] . Cinnamomum camphora (camphor laurel). CABI Compendium. CABI Publishing; 2019. doi: 10.1079/cabicompendium.13519

[21] Luo Q, Xu C, Zheng T, Ma Y, Li Y, Zuo Z. Leaf morphological and photosynthetic differences among four chemotypes of Cinnamomum camphora in different seasons. Industrial Crops and Products. 2021;169:113651. doi: 10.1016/j.indcrop.2021.113651

[22] Chen J, Tang C, Zhang R, Ye S, Zhao Z, Huang Y, et al. Metabolomics analysis to evaluate the antibacterial activity of the essential oil from the leaves of *Cinnamomum camphora* (Linn.) Presl. J Ethnopharmacol. 2020;253:112652. doi: 10.1016/j.jep.2020.112652 32035880

[23] Ni Z, Han X, Chen C, Zhong Y, Xu M, Xu L, et al. Integrating GC-MS and ssRNA-Seq analysis to identify long non-coding RNAs related to terpenoid biosynthesis in *Cinnamomum camphora*. Industrial Crops and Products. 2021;171:113875. doi: 10.1016/j.indcrop.2021.113875

[24] Muhamad SHA, On S, Sanusi SNA, Hashim AA, Addinna Zai MH. Antioxidant activity of Camphor leaves extract based on variation solvent. J Phys: Conf Ser. 2019;1349(1):012102. doi: 10.1088/1742-6596/1349/1/012102

[25] Lee HJ, Hyun E-A, Yoon WJ, Kim BH, Rhee MH, Kang HK, et al. In vitro anti-inflammatory and anti-oxidative effects of *Cinnamomum camphora* extracts. J Ethnopharmacol. 2006;103(2):208–16. doi: 10.1016/j.jep.2005.08.009 16182479

[26] Wang J, Su B, Jiang H, Cui N, Yu Z, Yang Y, et al. Traditional uses, phytochemistry and pharmacological activities of the genus *Cinnamomum (Lauraceae)*: A review. Fitoterapia. 2020;146:104675. doi: 10.1016/j.fitote.2020.104675 32561421

[27] Kang N-J, Han S-C, Yoon S-H, Sim J-Y, Maeng YH, Kang H-K, et al. *Cinnamomum camphora* Leaves Alleviate Allergic Skin Inflammatory Responses In Vitro and In Vivo. Toxicol Res. 2019;35(3):279–85. doi: 10.5487/TR.2019.35.3.279 31341557 PMC6629446

[28] Wang L, Zhang K, Zhang K, Zhang J, Fu J, Li J, et al. Antibacterial Activity of *Cinnamomum camphora Essential Oil* on Escherichia coli During Planktonic Growth and Biofilm Formation. Front Microbiol. 2020;11:561002. doi: 10.3389/fmicb.2020.561002 33304322 PMC7693543

[29] Wei C, Li H, Cui G, Ma C, Deng R, Zou Z, et al. Efficient separation of C*innamomum camphora* leaf essential oil and in vitro evaluation of its antifungal activity. Arabian Journal of Chemistry. 2022;15(11):104225. doi: 10.1016/j.arabjc.2022.104225

[30] *Cinnamomum camphora* (camphor laurel). CABI Compendium. CABI Publishing; 2019. doi: 10.1079/cabicompendium.13519

[31] Shahzad A, Parveen S, Sharma S, Shaheen A, Saeed T, Yadav V, et al. Plant Tissue Culture: Applications in Plant Improvement and Conservation. Plant Biotechnology: Principles and Applications. Springer Singapore. 2017. p. 37–72. doi: 10.1007/978-981-10-2961-5_2

[32] Samaniego I, Espin S, Cuesta X, Arias V, Rubio A, Llerena W, et al. Analysis of Environmental Conditions Effect in the Phytochemical Composition of Potato (*Solanum tuberosum*) Cultivars. Plants (Basel). 2020;9(7):815. doi: 10.3390/plants9070815 32610590 PMC7412447

[33] Lee ON, Ak G, Zengin G, Cziáky Z, Jekő J, Rengasamy KRR, et al. Phytochemical Composition, Antioxidant Capacity, and Enzyme Inhibitory Activity in Callus, Somaclonal Variant, and Normal Green Shoot Tissues of *Catharanthus roseus* (L) G. Don. Molecules. 2020;25(21):4945. doi: 10.3390/molecules25214945 33114628 PMC7663286

[34] Murashige T, Skoog F. A revised medium for rapid growth and bio assays with tobacco tissue cultures. J Plant Physiol. 1962;15(3):473–97.

[35] McCown BH, Lloyd G. Woody plant medium (WPM)—a mineral nutrient formulation for microculture of woody plant species. Hort Sci. 1981;16:453.

[36] Luck H. Catalase. In: Bergmeyer J, Grabi M, editors. Methods of Enzymatic Analysis. New York: Academic Press; 1974. p. 885–90.

[37] Beers RF Jr, Sizer IW. A spectrophotometric method for measuring the breakdown of hydrogen peroxide by catalase. Journal of Biological Chemistry. 1952;195(1):133–40. doi: 10.1016/s0021-9258(19)50881-x 14938361

[38] Maral J, Puget K, Michelson AM. Comparative study of superoxide dismutase, catalase and glutathione peroxidase levels in erythrocytes of different animals. Biochem Biophys Res Commun. 1977;77(4):1525–35. doi: 10.1016/s0006-291x(77)80151-4 901548

[39] Flohé L, Günzler WA. Assays of glutathione peroxidase. Methods Enzymol. 1984;105:114–21. doi: 10.1016/s0076-6879(84)05015-1 6727659

[40] Kyoung Chun O, Kim D-O. Consideration on equivalent chemicals in total phenolic assay of chlorogenic acid-rich plums. Food Research International. 2004;37(4):337–42. doi: 10.1016/j.foodres.2004.02.001

[41] Ivanova V, Stefova M, Vojnoski B, Dörnyei Á, Márk L, Dimovska V, et al. Identification of polyphenolic compounds in red and white grape varieties grown in R. Macedonia and changes of their content during ripening. Food Research International. 2011;44(9):2851–60. doi: 10.1016/j.foodres.2011.06.046

[42] Du L, Li Y, Yao Y, Zhang L. An Efficient Protocol for Plantlet Regeneration via Direct Organogenesis by Using Nodal Segments from Embryo-Cultured Seedlings of *Cinnamomum camphora* L. PLoS One. 2015;10(5):e0127215. doi: 10.1371/journal.pone.0127215 25962170 PMC4427331

[43] Goyal S, Chatterjee V, Kulkarni VM, Bhat V. Plant regeneration through somatic embryogenesis in cell suspensions of Cenchrus ciliaris L. Plant Methods. 2023;19(1):110. doi: 10.1186/s13007-023-01081-3 37853411 PMC10585800

[44] Hemmati N, Cheniany M, Ganjeali A. Effect of plant growth regulators and explants on callus induction and study of antioxidant potentials and phenolic metabolites in *Salvia tebesana* Bunge. Bot Serb. 2020;44(2):163–73. doi: 10.2298/botserb2002163h

[45] Mayerni R, Satria B, Wardhani D, Chan S. Effect of auxin (2,4-D) and cytokinin (BAP) in callus induction of local patchouli plants (*Pogostemon cablin Benth*.). IOP Conf Ser: Earth Environ Sci. 2020;583(1):012003. doi: 10.1088/1755-1315/583/1/012003

[46] Pakum W, Inmano O, Kongbangkerd A. TDZ and 2,4-D on in vitro propagation of panda plant from leaf explants. Ornam Hortic. 2021;27(1):41–8. doi: 10.1590/2447-536x.v27i1.2251

[47] Li Y, Zhao J, Chen H, Yu X, Li H, Zhang Y, et al. Plant regeneration via callus-mediated organogenesis in commercial variety of Chuanbeichai No. 1 in Bupleurum chinense DC. Plant Biotechnol Rep. 2023;17(2):159–69. doi: 10.1007/s11816-022-00772-y 35873882 PMC9288091

[48] Soulange JG, Ranghoo-Sa VM, Seeburrun SD. Tissue Culture and RAPD Analysis of *Cinnamomum camphora* and *Cinnamomum verum*. Biotechnology. 2007;6(2):239–44. doi: 10.3923/biotech.2007.239.244

[49] Kurup SS, Thayale Purayil F, Alkhaili MMS, Tawfik NH, Cheruth AJ, Kabshawi M, et al. Thidiazuron (TDZ) induced organogenesis and clonal fidelity studies in Haloxylon persicum (Bunge ex Boiss & Buhse): an endangered desert tree species. Physiol Mol Biol Plants. 2018;24(4):683–92. doi: 10.1007/s12298-018-0532-5 30042622 PMC6041233

[50] Huang L-C, Huang B-L, Murashige T. A micropropagation protocol forCinnamomum camphora. In Vitro CellDevBiol-Plant. 1998;34(2):141–6. doi: 10.1007/bf02822779

[51] Nirmal Babu K, Sajina A, Minoo D, John CZ, Mini PM, Tushar KV, et al. Micropropagation of camphor tree (Cinnamomum camphora). Plant Cell, Tissue and Organ Culture. 2003;74(2):179–83. doi: 10.1023/a:1023988110064

[52] Chen M, Ye Z, Ouyang S, Lin S, Shao A, Huang L. Callus induction of *Cinnamonum camphora* and formation of borneol. Zhongguo Zhong Yao Za Zhi. 2010;35(5):558–60. doi: 10.4268/cjcmm20100503 20506810

[53] Aref MS, Salem SS. Bio-callus synthesis of silver nanoparticles, characterization, and antibacterial activities via *Cinnamomum camphora* callus culture. Biocatalysis and Agricultural Biotechnology. 2020;27:101689. doi: 10.1016/j.bcab.2020.101689

[54] Hesami M, Daneshvar MH, Yoosefzadeh-Najafabadi M, Alizadeh M. Effect of plant growth regulators on indirect shoot organogenesis of Ficus religiosa through seedling derived petiole segments. J Genet Eng Biotechnol. 2018;16(1):175–80. doi: 10.1016/j.jgeb.2017.11.001 30647720 PMC6296569

[55] Muthi’ah A, Sakya AT, Setyawati A, Samanhudi Rahayu M. Callus induction of *Calotropis gigantea* using BAP and 2,4-D in vitro. IOP Conf Ser: Earth Environ Sci. 2023;1177(1):012021. doi: 10.1088/1755-1315/1177/1/012021

[56] Rybin DA, Sukhova AA, Syomin AA, Zdobnova TA, Berezina EV, Brilkina AA. Characteristics of Callus and Cell Suspension Cultures of Highbush Blueberry (*Vaccinium corymbosum* L.) Cultivated in the Presence of Different Concentrations of 2,4-D and BAP in a Nutrient Medium. Plants (Basel). 2024;13(23):3279. doi: 10.3390/plants13233279 39683072 PMC11644369

[57] Zakaria TNAAT, Tan HS, Hassan Z, Subramaniam S, Chew BL. Effects Of 2,4-D, BAP, and Sucrose Concentrations in The Callus Induction of White (*Clitoria ternatea var. Albiflora*) and Blue Butterfly Pea (*Clitoria ternatea*). MAB. 2024;53(4):53–63. doi: 10.55230/mabjournal.v53i4.3087

[58] Bong FJ, Yeou Chear NJ, Ramanathan S, Mohana-Kumaran N, Subramaniam S, Chew BL. The development of callus and cell suspension cultures of Sabah Snake Grass (*Clinacanthus nutans*) for the production of flavonoids and phenolics. Biocatalysis and Agricultural Biotechnology. 2021;33:101977. doi: 10.1016/j.bcab.2021.101977

[59] Lu X, Fei L, Li Y, Du J, Ma W, Huang H, et al. Effect of different plant growth regulators on callus and adventitious shoots induction, polysaccharides accumulation and antioxidant activity of *Rhodiola dumulosa*. Chin Herb Med. 2023;15(2):271–7. doi: 10.1016/j.chmed.2022.07.005 37265763 PMC10230626

[60] Al-Ajlouni ZI, Abbas S, Shatnawi M, Al-Makhadmeh I. In vitro propagation, callus induction, and evaluation of active compounds on *Ruta graveolens*. J Food Agricult Environ. 2015;13(2):101–6.

[61] S. A, A. G. Callus culture and in vitro production of anthraquinone in *Gynochthodes umbellata* (L.) Razafim. & B. Bremer (*Rubiaceae*). Industrial Crops and Products. 2017;95:608–14. doi: 10.1016/j.indcrop.2016.11.021

[62] Farhadi N, Panahandeh J, Azar AM, Salte SA. Effects of explant type, growth regulators and light intensity on callus induction and plant regeneration in four ecotypes of Persian shallot (*Allium hirtifolium*). Scientia Horticulturae. 2017;218:80–6. doi: 10.1016/j.scienta.2016.11.056

[63] Abu-Romman S, Suwwan M, Al-ramamneh EA-D. The influence of plant growth regulators on callus induction from hypocotyls of cucumber (*Cucumis sativus L.*). Advances in Environmental Biology. 2013;7:339–43.

[64] Yaroshko O, Pasternak T, Larriba E, Pérez-Pérez JM. Optimization of Callus Induction and Shoot Regeneration from Tomato Cotyledon Explants. Plants (Basel). 2023;12(16):2942. doi: 10.3390/plants12162942 37631154 PMC10459365

[65] Abd El-Kader EM, Serag A, Aref MS, Ewais EEA, Farag MA. Metabolomics reveals ionones upregulation in MeJA elicited *Cinnamomum camphora* (camphor tree) cell culture. Plant Cell Tiss Organ Cult. 2019;137(2):309–18. doi: 10.1007/s11240-019-01572-z

[66] Huang X, Liu J, Feng H, Ma Y, Zhang L, Han H. Effects of different plant hormones on callus induction and plant regeneration of miniature roses (*Rosa hybrida L.*). HIJ. 2018;2(4). doi: 10.15406/hij.2018.02.00053

[67] Marković M, Trifunović-Momčilov M, Radulović O, Paunović DM, Antonić Reljin DD, Uzelac B, et al. The Effects of Different Auxin–Cytokinin Combinations on Morphogenesis of Fritillaria meleagris Using Bulb Scale Sections In Vitro. Horticulturae. 2023;9(8):910. doi: 10.3390/horticulturae9080910

[68] Klimaszewska K, Hargreaves C, Lelu-Walter M-A, Trontin J-F. Advances in Conifer Somatic Embryogenesis Since Year 2000. Methods Mol Biol. 2016;1359:131–66. doi: 10.1007/978-1-4939-3061-6_7 26619862

[69] Junairiah Mahmuda A, Manuhara YSW, Ni’matuzahroh Sulistyorini L. Callus Induction and Bioactive Compounds from *Piper betleL*. var nigra. IOP Conf Ser: Earth Environ Sci. 2019;217:012026. doi: 10.1088/1755-1315/217/1/012026

[70] Astuti RD, Harahap F, Edi S. Callus Induction of Mangosteen (*Garcinia mangostana* L.) In Vitro with Addition of Growth Regulators. J Phys: Conf Ser. 2020;1485(1):012029. doi: 10.1088/1742-6596/1485/1/012029

[71] Fazal H, Abbasi BH, Ahmad N, Ali M. Elicitation of Medicinally Important Antioxidant Secondary Metabolites with Silver and Gold Nanoparticles in Callus Cultures of Prunella vulgaris L. Appl Biochem Biotechnol. 2016;180(6):1076–92. doi: 10.1007/s12010-016-2153-1 27287999

[72] Efferth T. Biotechnology Applications of Plant Callus Cultures. Engineering. 2019;5(1):50–9. doi: 10.1016/j.eng.2018.11.006

[73] Ali AYA, Ibrahim MEH, Zhou G, Nimir NEA, Elsiddig AMI, Jiao X, et al. Gibberellic acid and nitrogen efficiently protect early seedlings growth stage from salt stress damage in Sorghum. Sci Rep. 2021;11(1):6672. doi: 10.1038/s41598-021-84713-9 33758238 PMC7988071

[74] Liang X, Chen Q, Lu H, Wu C, Lu F, Tang J. Increased activities of peroxidase and polyphenol oxidase enhance cassava resistance to Tetranychus urticae. Exp Appl Acarol. 2017;71(3):195–209. doi: 10.1007/s10493-017-0125-y 28405840

[75] Li Z-X, Lan J-B, Liu Y-Q, Qi L-W, Tang J-M. Investigation of the role of AcTPR2 in kiwifruit and its response to Botrytis cinerea infection. BMC Plant Biol. 2020;20(1):557. doi: 10.1186/s12870-020-02773-x 33302873 PMC7731759

[76] Muchtaridi M, Az-Zahra F, Wongso H, Setyawati LU, Novitasari D, Ikram EHK. Molecular Mechanism of Natural Food Antioxidants to Regulate ROS in Treating Cancer: A Review. Antioxidants (Basel). 2024;13(2):207. doi: 10.3390/antiox13020207 38397805 PMC10885946

[77] Sharma H. In vitro propagation using nodal explants of *Cinnamomum camphora*: an important medicinal tree. Inter J Res Biosci Agricult Technol. 2020;17: 394–401.

[78] Rattanawong K, Koiso N, Toda E, Kinoshita A, Tanaka M, Tsuji H, et al. Regulatory functions of ROS dynamics via glutathione metabolism and glutathione peroxidase activity in developing rice zygote. Plant J. 2021;108(4):1097–115. doi: 10.1111/tpj.15497 34538012 PMC9293154

[79] Sachdev S, Ansari SA, Ansari MI, Fujita M, Hasanuzzaman M. Abiotic Stress and Reactive Oxygen Species: Generation, Signaling, and Defense Mechanisms. Antioxidants (Basel). 2021;10(2):277. doi: 10.3390/antiox10020277 33670123 PMC7916865

[80] Passaia G, Margis-Pinheiro M. Glutathione peroxidases as redox sensor proteins in plant cells. Plant Sci. 2015;234:22–6. doi: 10.1016/j.plantsci.2015.01.017 25804806

[81] Madhu Sharma A, Kaur A, Tyagi S, Upadhyay SK. Glutathione Peroxidases in Plants: Innumerable Role in Abiotic Stress Tolerance and Plant Development. J Plant Growth Regul. 2022;42(2):598–613. doi: 10.1007/s00344-022-10601-9

[82] Strycharz-Dudziak M, Fołtyn S, Dworzański J, Kiełczykowska M, Malm M, Drop B, et al. Glutathione Peroxidase (GPx) and Superoxide Dismutase (SOD) in Oropharyngeal Cancer Associated with EBV and HPV Coinfection. Viruses. 2020;12(9):1008. doi: 10.3390/v12091008 32917014 PMC7551554

[83] Guruprasad Y, Gurudath S, Naik R, Ganapathy K, Sujatha D, Pai A. Superoxide dismutase and glutathione peroxidase in oral submucous fibrosis, oral leukoplakia, and oral cancer: A comparative study. J Orofac Sci. 2012;4(2):114. doi: 10.4103/0975-8844.106202 23167351

[84] Khan T, Abbasi BH, Khan MA, Shinwari ZK. Differential Effects of Thidiazuron on Production of Anticancer Phenolic Compounds in Callus Cultures of *Fagonia indica*. Appl Biochem Biotechnol. 2016;179(1):46–58. doi: 10.1007/s12010-016-1978-y 26758711

[85] Nagai N, Kitauchi F, Okamoto K, Kanda T, Shimosaka M, Okazaki M. A transient increase of phenylalanine ammonia-lyase transcript in kinetin-treated tobacco callus. Biosci Biotechnol Biochem. 1994;58(3):558–9. doi: 10.1271/bbb.58.558 7513564

[86] Reshi ZA, Ahmad W, Lukatkin AS, Javed SB. From Nature to Lab: A Review of Secondary Metabolite Biosynthetic Pathways, Environmental Influences, and In Vitro Approaches. Metabolites. 2023;13(8):895. doi: 10.3390/metabo13080895 37623839 PMC10456650

[87] Pérez-Tortosa V, López-Orenes A, Martínez-Pérez A, Ferrer MA, Calderón AA. Antioxidant activity and rosmarinic acid changes in salicylic acid-treated *Thymus membranaceus* shoots. Food Chemistry. 2012;130(2):362–9. doi: 10.1016/j.foodchem.2011.07.051

[88] Hatami M, Kariman K, Ghorbanpour M. Engineered nanomaterial-mediated changes in the metabolism of terrestrial plants. Sci Total Environ. 2016;571:275–91. doi: 10.1016/j.scitotenv.2016.07.184 27485129

